# Bacteria Causing Community-Acquired Urinary Tract Infections and Their Antibiotic Susceptibility Patterns in Outpatients Attending at a State Hospital in Turkey

**DOI:** 10.7759/cureus.17753

**Published:** 2021-09-06

**Authors:** Reyhan Öztürk, Gokhan Tazegul

**Affiliations:** 1 Infectious Diseases, Ankara Polatlı Duatepe State Hospital, Ankara, TUR; 2 Internal Medicine, Ankara Polatlı Duatepe State Hospital, Ankara, TUR

**Keywords:** fosfomycin, bacteriuria, gentamicin, nitrofurantoin, urinary tract infections

## Abstract

Introduction

Clinicians should know the frequency and resistance patterns of bacteria that cause urinary tract infections (UTI) to provide patients with appropriate treatment and antibiotic management. However, the frequency of culture reproducing organisms and resistance patterns change in each community. Therefore, these data must be determined locally to make better treatment decisions. Herein, we aimed to determine the frequency of UTI-causing agents and current antimicrobial resistance profiles in outpatients attending our hospital.

Methods

This retrospective descriptive study included three hundred eight outpatients attending under the diagnosis of UTI between March and October 2020 who had a positive urine culture for bacterial growth. Age, sex, laboratory tests, urinalysis results, microorganisms grown in urine culture, and antibiograms were evaluated from the patients' medical records. Data were analyzed using SPSS version 23.0 (IBM Corp., Armonk, NY) for Windows.

Results

In urine culture results, *Escherichia coli* (*E. coli)* and *Klebsiella* species are the most commonly detected agents. The growth in 71 (23%) of the 308 cultures was extended-spectrum beta-lactamase (ESBL) positive. In the *E. coli* growths, the susceptibility rates to fosfomycin, gentamicin, nitrofurantoin, trimethoprim-sulfamethoxazole, and ampicillin were 95.2%, 90.3%, 95.3%, 76.8%, and 49.3%, respectively. The susceptibility of *Klebsiella *species to gentamicin was as high as 93.7%, similar to that of *E. coli*, whereas its susceptibility rates to fosfomycin, trimethoprim-sulfamethoxazole, and nitrofurantoin were lower than those of *E. coli* (76.1%, 48.4%, and 68.4%, respectively). Of the 71 ESBL-positive growths, 52 were *E. coli* (17.3% of all UTIs), and 14 were *Klebsiella *species (4.6% of all UTIs). Of the ESBL-positive strains, 88.7%, 81%, and 76.1% were susceptible to fosfomycin and nitrofurantoin, respectively, and 64.9% and 45.7% were sensitive to cefoxitin and trimethoprim-sulfamethoxazole.

Conclusion

UTIs are among the most common causes of hospital admission and infections for which empirical antibiotic administration is initiated. The increasing rates of ESBL positivity and resistance to antibiotics such as ampicillin, cephalosporins, trimethoprim-sulfamethoxazole, and quinolones, especially in *E. coli *and *Klebsiella* strains, which are the most common pathological agents of UTI in our region, have limited the use of these treatments. However, the high susceptibility of *E. Coli* growths to fosfomycin and nitrofurantoin and susceptibility of *Klebsiella* growths to gentamicin may make these antibiotics stand out as suitable options for the empirical treatment of UTI in our setting.

## Introduction

Urinary tract infections (UTIs) are among the most common bacterial infections encountered in the community and hospitals [1,2]. UTI is defined as the coexistence of urinary tract symptoms and bacteriuria [3,4]. However, in almost all cases of community-acquired UTI, antibiotic therapy is prescribed empirically before final urine culture or other laboratory results are obtained [2]. Over the years, the frequency of fluoroquinolone-resistant pathogens has increased owing to the increased empirical use of fluoroquinolones, frequency of gram-negative bacteria such as *Escherichia coli* and *Klebsiella* that produce extended-spectrum beta-lactamase (ESBL), and frequency of *Enterobacteriaceae* with multiple resistance mechanisms, including carbapenemase, which influences decisions regarding the empirical treatment of UTI [2,5,6].

Therefore, clinicians should know the frequency and resistance patterns of bacteria that cause UTI to provide patients with appropriate treatment and antibiotic management. The reported data in the literature are conflicting regarding the antimicrobial susceptibility patterns of UTI-causing organisms [7]. Therefore, the clinicians must determine the culture results and antibiotic resistance patterns of the reproducing agents in UTI cases locally to make better antibiotic therapy decisions and prevent the development of antibiotic resistance in the community. This study aimed to determine the frequency of UTI-causing agents and current antimicrobial resistance profiles in outpatients attending our hospital.

## Materials and methods

This retrospective descriptive study was conducted per the Declaration of Helsinki by obtaining data use permission from the Ankara Polatlı Duatepe State Hospital Administration. The researchers were provided with fully anonymized data for the study by the hospital. Ankara Research and Training Hospital Ethics Committee provided ethical approval for this study (Approval number: E-21-672, Approval date: 18.08.2021).

Three hundred eight outpatients with the diagnosis of UTI between March and October 2020 who had a positive urine culture for bacterial growth were included in our study. Age, sex, white blood cell (WBC) count, and C-reactive protein (CRP) levels from laboratory tests, urinalysis results, microorganisms grown in urine culture, and antibiograms were evaluated from the medical records of the patients included in the study. WBC count was measured using the automated cell counter ABX Pentra DF 120 (Horiba Medical, Japan). CRP levels were analyzed with the AU5800 autoanalyzer (Beckman Coulter, Brea, CA) using the immunoturbidimetric method. Chemical examinations for urinalysis were routinely performed with BT Uricell 1600 (BT Products, Turkey), and microscopic examinations were performed with BT Uricell 1280 (BT Products, Turkey). pH, red blood cell (RBC) count per high power field (HPF), and nitrite, leukocyte esterase, protein, and glucose levels were evaluated from the urinalysis. Microscopic findings of >10 WBC per HPF in urine samples were considered significant for UTI [8].

For urine culture, midstream urine samples were routinely collected from the patients after appropriate disinfection practices. After midstream urine samples were collected, 10 mL of urine was taken within 1 hour, inoculated with a round loop to a blood and Eosin Methylene Blue (EMB) agar medium, and incubated at 37°C for 24 hours in an oxygen-stable and suitable humid environment. The culture was checked for bacterial growth the next day. Bacterial identification and an antibiogram test using the cultures were performed with VITEK 2 Compact (bioMérieux SA, Marcy l'Etoile, France). Bacterial growth of ≥105 colony-forming units (CFU)/mL in the culture was considered significant. Antibiograms have not been studied for smaller growths. Contamination is considered when more than two different types of growth are present.

The antibiotics evaluated for susceptibility using the minimal inhibitory concentration technique were as follows: fosfomycin, gentamicin, cefixime, cefuroxime, ciprofloxacin, ampicillin, trimethoprim-sulfamethoxazole, cefoxitin, nitrofurantoin, ceftazidime, piperacillin-tazobactam, meropenem, and ertapenem for gram-negative microorganisms; amikacin, levofloxacin, ceftriaxone, netilmicin, cefepime, tobramycin, and aztreonam for gram-negative growths; and teicoplanin, vancomycin, tigecycline, linezolid, co-amoxiclav, penicillin, colistin, clindamycin, tetracycline, and daptomycin for gram-positive growths. When available, antibiograms have been routinely studied with the following antibiotics under minimal conditions: fosfomycin, gentamicin, cefixime, cefuroxime, ciprofloxacin, ampicillin, trimethoprim-sulfamethoxazole, cefoxitin, and nitrofurantoin. Other antibiotics were added to the antibiogram as deemed appropriate by the laboratory.

Data were analyzed using SPSS version 23.0 (IBM Corp., Armonk, NY) for Windows. Continuous variables were expressed as median (interquartile [IQR] range), and categorical data as values and percentages. In the comparative analysis, chi-square tests were used for categorical variables, and the Mann-Whitney U test was used for continuous variables. For all the statistical tests, p values < 0.05 were accepted as the statistical significance limit.

## Results

Of the 308 outpatients included in the study, 220 (71.4%) were female, and 88 (28.6%) were male. The median age of the patients was 41 years (IQR, 19-69 years). We did not include eight cultures (2.6%) in the antibiogram because the bacterial growths were considered contaminated owing to the growth of a low amount of microorganisms or >2 kinds of microorganisms. In urine culture results, *E. coli* and *Klebsiella* species are the most commonly detected agents. The frequency rates of all the agents are shown in Table 1. The serum CRP and WBC measurements and complete urinalysis results are shown in Table 2.

**Table 1:** Distribution of bacterial growths in urine cultures

Bacterial growth	n (%)
Escherichia coli	221 (71.7)
*Klebsiella* species	33 (10.7)
*Staphylococcus *species	16 (5.2)
*Enterococcus *species	9 (2.9)
Proteus mirabilis	6 (1.9)
Enterobacter cloaca	5 (1.6)
*Pseudomonas* species	4 (1.3)
*Streptococcus* species	3 (1)
*Acinetobacter* species	2 (0.6)
*Citrobacter* species	1 (0.3)
Not included in antibiograms	8 (2.8)

**Table 2:** Serum white blood cell counts, C-reactive protein levels, and urinalysis results of the study group IQR: Interquartile range, RBC: Red blood cell, WBC: White blood cell, CRP: C-reactive protein, HPF: high power field.

		Median (IQR)	(−) n (%)	(+) n (%)	(++) n (%)	(+++) n (%)	(++++) n (%)
Blood tests							
	WBC (per mm^3^)	7500 (6200-9400)	-	-	-	-	-
	CRP (mg/dL) (Normal value: less than 0.5 mg/dL)	0.69 (0.26-4.05)	-	-	-	-	-
Urinalysis							
	pH	5.5(1)	-	-	-	-	-
	RBC per HPF	5(17)	-	-	-	-	-
	Nitrite	-	208 (67.5)	100 (32.5)			
	Leukocyte Esterase	-	75 (24.4)	91 (29.5)	50 (16.2)	89 (28.9)	3 (1)
	Protein	-	244 (79.2)	53 (17.2)	10 (3.2)	1 (0.3)	-
	Glucose	-	292 (94.8)	5 (1.6)	1 (0.3)	3 (1)	7 (2.3)

We evaluated gram-negative growths comparatively in three groups, namely *E. coli, Klebsiella* species, and other gram-negative bacteria. We did not perform subgroup analysis on the gram-positive growths because of their small number. When we compared the demographic and laboratory data according to pathogens, the median ages of the patients with *E. coli, Klebsiella* species, and other gram-negative growths were 40 years (IQR, 20-65 years), 58 years (IQR, 40-77 years), and 69 years (IQR, 8-74 years), respectively. The median ages of the patients with* Klebsiella *and *E. coli* overgrowths were significantly different (Mann-Whitney U test, p = 0.005). The proportions of female patients were 77.8% (n = 172), 51.5% (n = 17), and 50% (n = 9) among the patients with *E. coli, Klebsiella,* and other gram-negative growths, respectively (chi-square test, p = 0.0001). No significant differences in serum white blood cell count, CRP level, urine pH, RBC count, nitrite, bacteria, leukocyte esterase, protein, and glucose measurements were found between patient groups divided according to the bacterial growths in their urine cultures.

The antibiotic susceptibility of the microorganisms grown in the urine culture is shown in Table 3. In the *E. coli* growths, the susceptibility rates to fosfomycin, gentamicin, nitrofurantoin, trimethoprim-sulfamethoxazole, and ampicillin were 95.2%, 90.3%, 95.3%, 76.8%, and 49.3%, respectively. The susceptibility of* Klebsiella* species to gentamicin was as high as 93.7%, similar to that of *E. coli*, whereas its susceptibility rates to fosfomycin, trimethoprim-sulfamethoxazole, and nitrofurantoin were lower than those of *E. coli* (76.1%, 48.4%, and 68.4%, respectively). The rates of sensitivity of the *Klebsiella* and *Proteus *species to ampicillin were 11.1% and 50%, respectively. In the antibiograms of *Staphylococcus* species, the third most common growth, the sensitivity to trimethoprim-sulfamethoxazole was 92.8%, and to vancomycin and tigecycline was 100%. The sensitivity to nitrofurantoin and ampicillin was also 100%; however, the number of antibiograms was low.

**Table 3:** Antibiogram results according to the growths in the urine cultures Data were presented as values of susceptible growth divided by values of all antibiograms and % susceptible.

Drug	*E. coli* (n=221)	*Klebsiella *species (n=33)	*Staphylococcus* species (n=16)	*Enterococcus *species (n=9)	*Proteus* species (n=6)	*Enterobacter cloaca* (n=5)	*Pseudomonas *species (n=4)	*Streptococcus *species (n=3)	*Acinetobacter *species (n=2)	*Citrobacter* species (n=1)
Fosfomycin	201/211 (95.2%)	16/21 (76.1%)	4/11 (36.3%)	-	5/5 (100%)	3/4 (75%)	-	-	-	-
Gentamicin	197/218 (90.3%)	30/32 (93.7%)	13/15 (86.6%)	5/6 (83.3%)	5/6 (83.3%)	3/5 (60%)	3/4 (75%)	2/2 (100%)	2/2 (100%)	1/1 (100%)
Cefixime	143/211 (68.1%)	14/32 (43.7%)	1/1 (100%)	-	4/6 (66.7%)	0/5 (0%)	-	-	-	1/1 (100%)
Cefuroxime	123/182 (67.5%)	13/30 (43.3%)	1/1 (100%)	-	4/6 (66.7%)	0/4 (0%)	-	-	-	-
Ciprofloxacin	159/207 (77.1%)	13/28 (46.4%)	10/14 (71.4%)	4/7 (57.1%)	6/6 (100%)	1/5 (20%)	2/4 (50%)	-	0/2 (0%)	1/1 (100%)
Ampicillin	75/152 (49.3%)	2/18 (11.1%)	1/1 (100%)	6/8 (75%)	2/4 (50%)	0/4 (0%)	-	-	-	1/1 (100%)
Trimethoprim-Sulfamethoxazole	166/216 (76.8%)	16/33 (48.4%)	13/14 (92.8%)	0/2 (0%)	5/6 (83.3%)	2/5 (40%)	-	2/2 (100%)	0/2 (0%)	1/1 (100%)
Cefoxitin	102/119 (85.7%)	11/14 (78.5%)	6/12 (50%)	-	2/3 (66.7%)	1/3 (33.3%)	-	-	-	-
Nitrofurantoin	184/193 (95.3%)	13/19 (68.4%)	4/4 (100%)	1/1 (100%)	2/4 (50%)	1/3 (33.3%)	-	-	-	-
Ceftazidime	11/66 (16.6%)	0/14 (0%)	-	-	2/4 (50%)	0/3 (0%)	3/4 (75%)	-	0/1 (0%)	-
Piperacillin Tazobactam	40/66 (64.5%)	7/16 (43.7%)	-	-	2/2 (100%)	0/4 (0%)	2/4 (50%)	-	-	-
Meropenem	20/22 (90.9%)	12/12 (100%)	-	-	2/2 (100%)	3/3 (100%)	0/2 (0%)	-	0/1 (0%)	-
Ertapenem	16/19 (84.2%)	9/9 (100%)	-	-	2/2 (100%)	3/4 (75%)	-	-	-	-
İmipenem	18/21 (85.7%)	14/14 (100%)	-	-	-	4/4 (100%)	0/2 (0%)	-	0/2 (0%)	-
Amikacin	30/37 (81%)	8/9 (88.8%)	-	-	3/3 (100%)	2/2 (100%)	0/2 (0%)	-	0/2 (0%)	-
Levofloxacin	1/7 (14.2%)	0/1 (0%)	0/1 (0%)	-	-	-	1/2 (50%)	-	-	-
Teicoplanin	0/1 (0%)	-	3/4 (75%)	1/2 (50%)	-	-	-	-	-	-
Vancomycin	-	-	7/7 (100%)	1/2 (50%)	-	-	-	-	-	-
Tigecycline	2/2 (100%)	2/3 (66.6%)	6/6 (100%)	3/3 (100%)	-	-	-	-	-	-
Linezolid	3/3 (100%)	-	6/6 (100%)	3/3 (100%)	-	-	-	-	-	-
Ceftriaxone	5/64 (7.8%)	0/17 (0%)	-	-	0/2 (0%)	0/4 (0%)	-	-	-	-
Amoxicillin clavulanate	8/9 (88.8%)	1/2 (50%)	-	-	1/1 (100%)	-	-	-	-	-
Penicillin	0/1 (0%)	-	3/3 (100%)	-	-	-	-	3/3 (100%)	-	-
Colistin	1/1 (100%)	-	-	-	-	-	1/1 (100%)	-	-	-
Aztreonam	1/2 (50%)	-	-	-	-	-	0/1 (0%)	-	-	-
Netilmicin	0/2 (0%)	0/1 (0%)	-	-	-	-	-	-	-	-
Clindamycin	2/3 (66.6%)	-	7/12 (58.3%)	-	-	-	-	-	-	-
Tetracycline	1/2 (50%)	-	4/11 (36.3%)	-	-	-	-	1/2 (50%)	-	-
Cefepime	0/5 (0%)	0/1 (0%)	-	-	-	-	-	-	-	-
Tobramycin	0/2 (0%)	0/1 (0%)	-	-	-	-	-	-	-	-
Daptomycin	2/2 (100%)	-	6/6 (100%)	-	-	-	-	-	-	-

A gram-negative antibiogram panel susceptibility comparison between *E. coli, Klebsiella *species, and other gram-negative strains is presented in Table 4. The susceptibility rates to fosfomycin, cefixime, cefuroxime, ampicillin, and trimethoprim-sulfamethoxazole were similar between other gram-negative growths and* E. coli* growths, but were higher than those of *Klebsiella* species. The rates of susceptibility of the *E. coli* growths to ciprofloxacin and nitrofurantoin were higher than those of other gram-negative growths and *Klebsiella* species growths. A significant difference in cefoxitin susceptibility was only present between *E. coli* and the other gram-negative growths. The susceptibility to gentamicin was similar between the three groups.

**Table 4:** Gram-negative antibiogram panel susceptibility comparison of E. coli, Klebsiella species, and other gram-negative strains Data were presented as values and percentages. Chi-square tests were used for categorical variables was used for analysis.

		*E. coli* (n=221)	*Klebsiella* species (n=33)	Other gram-negative bacteria (n=18)	P value
Fosfomycin	Susceptible	201 (95.2%)	16 (76.1%)	8 (88.9%)	
	Resistant	10 (4.8%)	5 (23.9%)	1 (11.1%)	0.003
Gentamicin	Susceptible	197 (90.3%)	30 (93.7%)	14 (77.8%)	
	Resistant	21 (9.7%)	2 (6.3%)	4 (22.2%)	0.17
Cefixime	Susceptible	143 (68.1%)	14 (43.7%)	5 (41.7%)	
	Resistant	67 (31.9%)	18 (56.3%)	7 (58.3%)	0.007
Cefuroxime	Susceptible	123 (67.6%)	13 (43.3%)	4 (40%)	
	Resistant	59 (32.5%)	17 (56.7)	6 (60%)	0.012
Ciprofloxacin	Susceptible	159 (77.1%)	13 (46.4%)	10 (55.6%)	
	Resistant	47 (22.9%)	15 (53.6%)	8 (44.4%)	0.001
Ampicilin	Susceptible	75 (49.3%)	2 (11.1%)	3 (33.3%)	
	Resistant	77 (50.7%)	16 (88.9%)	6 (66.7%)	0.007
Trimethoprim-Sulfamethoxazole	Susceptible	166 (76.8%)	16 (48.4%)	8 (57.1%)	
	Resistant	50 (23.2%)	17 (51.6%)	6 (42.9%)	0.001
Cefoxitin	Susceptible	102 (85.7%)	11 (78.5%)	3 (50%)	
	Resistant	17 (14.3%)	3 (21.5%)	3 (50%)	0.06
Nitrofurantoin	Susceptible	184 (95.3%)	13 (68.4%)	3 (42.9%)	
	Resistant	9 (4.7%)	6 (31.6%)	4 (57.1%)	0.0001

The growth in 71 (23%) of the 308 cultures was ESBL positive. Of the 71 ESBL-positive growths, 52 were *E. coli *(17.3% of all UTIs), 14 were *Klebsiella* species (4.6% of all UTIs), 3 were *Enterobacter* species (1% of all UTIs), and 2 were *Proteus* species (0.66% of all UTIs). The ESBL positivity rate was 23.3% in *E. coli,* 42.4% in *Klebsiella* species, and 10.9% in other growths. Carbapenemase was positive only in one *E. coli* culture. This strain was resistant to carbapenems and piperacillin-tazobactam but susceptible to fosfomycin, gentamicin, trimethoprim-sulfamethoxazole, nitrofurantoin, and ciprofloxacin. The ESBL positivity rate was similar between *Klebsiella *and *E. coli* (p = 0.21, chi-square test).

The median age was 39 years (IQR, 21-67 years) in the patients with ESBL-negative growth and was 57 years (IQR, 24-76 years) in those with ESBL-positive growth (Mann-Whitney U test, p = 0.047). The proportion of women was 74.7% (n = 171) among the patients with ESBL-negative culture growths and 59.2% (n = 42) among those with ESBL-positive growth (p = 0.01, chi-square test). We found no significant differences between the two groups regarding serum WBC count, CRP level, urine pH, RBC count, nitrite, leukocyte esterase, protein, and glucose measurements.

The gram-negative antibiogram panel susceptibility comparison of the ESBL-positive and ESBL-negative strains is shown in Table 5. The ampicillin, cefixime, and cefuroxime resistance rates were 100%, 98.6%, and 96.5%, respectively, in the ESBL-positive strains. Of the ESBL-positive strains, 88.7%, 81%, and 76.1%, were susceptible to fosfomycin and nitrofurantoin, respectively, and 64.9% and 45.7% were sensitive to cefoxitin and trimethoprim-sulfamethoxazole, respectively.

**Table 5:** Gram-negative antibiogram panel susceptibility comparison of ESBL-negative and ESBL-positive gram-negative strains Data were presented as values and percentages. Chi-square tests were used for categorical variables was used for analysis.

		ESBL negative (n=201)	ESBL positive (n=71)	P value
Fosfomycin	Susceptible	170 (95%)	55 (88.7%)	
	Resistant	9 (5%)	7 (11.3%)	0.08
Gentamicin	Susceptible	187 (94.9%)	54 (76.1%)	
	Resistant	10 (5.1%)	17 (23.9%)	0.0001
Cefixime	Susceptible	161 (87.5%)	1 (1.4%)	
	Resistant	23 (12.5%)	69 (98.6%)	0.0001
Cefuroxime	Susceptible	138 (83.6%)	2 (3.5%)	
	Resistant	27 (16.4%)	55 (96.5%)	0.0001
Ciprofloxacin	Susceptible	159 (83.7%)	23 (37.1%)	
	Resistant	31 (16.3%)	39 (62.9%)	0.0001
Ampicilin	Susceptible	80 (60.2%)	0 (0%)	
	Resistant	53 (39.8%)	46 (100%)	0.0001
Trimethoprim- Sulfamethoxazole	Susceptible	158 (81.9%)	32 (45.7%)	
	Resistant	35 (18.1%)	38 (54.3%)	0.0001
Cefoxitin	Susceptible	92 (90.2%)	24 (64.9%)	
	Resistant	10 (9.8%)	13 (35.1%)	0.001
Nitrofurantoin	Susceptible	153 (95%)	47 (81%)	
	Resistant	8 (5%)	11 (19%)	0.002

## Discussion

In cases of community-acquired UTI, antibiotic therapy is often prescribed before culture and susceptibility studies. To prescribe the appropriate antibiotic therapy for patients and reduce the development of antibiotic resistance, clinicians must determine the culture results and antibiotic resistance patterns of the agents grown in cultures locally. In this study, we aimed to determine the frequency and current antimicrobial resistance profiles of agents causing community-acquired UTI in outpatients attending our hospital, a 300-bed secondary care hospital in Turkey.

In our study, *E. coli* and *Klebsiella* species were the most common growths detected. Although *E. coli* is reported to be the most common cause of UTI in the literature, similar to our study, the second most common pathological agent differed between studies. Ağca reported that the second most common urine growths after *E. coli *were *Pseudomonas aeruginosa* (6%), *Klebsiella *species (5%), *Enterococcus *species (5%), and *Staphylococcus aureus* (4%) [9]. In another study conducted in Kosovo, the second most commonly isolated pathological agent was the *Proteus* species [10]. In two other studies from Turkey, *Klebsiella* species growths were reported as the second most common, similar to our finding [11,12]. Kidwai et al. reported* S. aureus *and *Klebsiella* species as the second most common growths after *E. coli* in patients in low socioeconomic strata [13].

Female sex has been reported as a risk factor of UTI in the literature. UTI occurs twice more frequently in women than in men [1,13]. The short urethra and proximity of the urethra to the anus have been reported to be among the factors that increase the risk of UTI [14]. Although we did not aim to determine the prevalence in our study, we found that 71.4% of the patients with urine culture growth were female. Moreover, the microorganisms reproduced in the culture of patients with UTI may also be related to sex. Similar to our results, previous studies reported that the proportion of females was higher among patients with *E. coli *growths, and the male-to-female ratio was close to 1 among patients with *Klebsiella *species growths [12].

Although studies on the antibiotic resistance pattern of all gram-negative growths are limited in the literature, studies on the growth of* E. coli* and *Klebsiella* species have been conducted in different centers. The susceptibility of *E. coli *strains to ampicillin has been reported to range from 11.6% to 28% [12,15], and its susceptibility to fosfomycin ranged from 60% to 98% [7,13,16]. On the other hand, the sensitivity to nitrofurantoin was found to be high in many studies (86.45%-94%) but low (59%) in the study of Kidwai et al. [1,7,8,13,15,16]. While the sensitivity to gentamicin was reported to be as low as 37%-45.65% in some studies [8,13], it was reported to be higher by Daoud et al. and Dash et al., similar to our results (91.3% and 94.1%, respectively) [1,16]. The sensitivity to trimethoprim-sulfamethoxazole ranged from 42.99% to 71.9% [1,7,8,15,16], while that to ciprofloxacin ranged from 68% to 91% [2]. In a study conducted in Turkey, high resistance to ciprofloxacin (22.1%) and cephalosporins (cefepime, 23.5%; ceftazidime, 22.5%; and ceftriaxone, 26.3%) was found in outpatients with *E. coli *growth in cultures, whereas lower resistance to amoxicillin-clavulanate (16.4%) and nitrofurantoin (4.7%) was observed [11]. In our study, the sensitivity of *E. coli* strains to ampicillin was 49.3%, higher than those reported in the literature. High susceptibility to fosfomycin, nitrofurantoin, and gentamicin was observed (95.2%, 95.3%, and 90.3%, respectively). While the susceptibility rate to trimethoprim-sulfamethoxazole was lower (76.8%), it was higher than those reported in other studies. The rates of sensitivity to ciprofloxacin and cephalosporins were similar to those reported in the literature.

In studies that evaluated the antibiotic susceptibility of *Klebsiella *strains, Shaifali et al. reported an ampicillin susceptibility rate of 54.54% [8]. In the literature, the rates of susceptibility to fosfomycin, gentamicin, nitrofurantoin, trimethoprim-sulfamethoxazole, and ciprofloxacin were 53%, 50%-54%, 25%-90%, 47%-81%, 53%-100%, respectively [2,8,13,15]. Rizvi et al. reported a 100% rate of susceptibility to fosfomycin [7]. In our study, the susceptibility rate of the *Klebsiella *species growth to ampicillin was much lower than those reported in the literature. Similar to the susceptibility of *E. coli, *the susceptibility of the *Klebsiella *species to gentamicin was higher (93.7%). The rates of susceptibility of the *Klebsiella *species to fosfomycin, nitrofurantoin, trimethoprim-sulfamethoxazole, and ciprofloxacin were lower than those of E. coli (76.1%, 68.4%, 48.4%, and 46.4%, respectively) and similar to those reported in other studies.

As the ESBL positivity rates vary among hospitals and regions, hospitals must conduct surveillance studies to determine the ESBL positivity rates and resistance patterns. In different studies from Turkey, the ESBL positivity rates ranged from 7.2% to 53% for *E. coli *strains and from 32% to 54% for *Klebsiella *species growths [17-20]. In our study, the ESBL positivity rate was 23.3% for the *E. coli* strains, 42.4% for the *Klebsiella *species, and 10.9% for other gram-negative growths. Carbapenemase production was detected in one *E. coli *strain. These findings are similar to those reported in the literature, and our ESBL positivity rate may be similar to that in large-center hospitals, as our hospital is a 300-bed secondary care hospital with 30 intensive care beds and a center serving patients from different regions.

The antibiotic susceptibility rates of ESBL-positive microorganisms also differed in the literature. In a study conducted in Tunisia, the rates of susceptibility of ESBL-positive* E. coli* strains to fosfomycin, nitrofurantoin, and trimethoprim-sulfamethoxazole were 100%, 96.4%, and 36.4%, respectively, and the rate of sensitivity of ESBL-positive *E. coli* strains to ciprofloxacin was 38.1% [16]. In our study, the rate of susceptibility of ESBL-positive strains was 88.7% for fosfomycin, 81% for nitrofurantoin, 45.7% for trimethoprim-sulfamethoxazole, and 77.1% for ciprofloxacin. This difference may be due to the differences in ESBL genes and antibiotics used by the patient populations in the previous studies.

Our results should be interpreted with consideration of the limitations of this study. Owing to the study's retrospective design and limited data available in the electronic medical records in our hospital, we could not obtain information on individual patient history regarding risk factors such as urinary stones, urinary catheterization, or other instrumentations. In addition, other known risk factors of UTI (e.g., diabetes) were not considered; however, investigating these factors is beyond the scope of our study.

## Conclusions

UTIs are among the most common causes of hospital admission and infections for which empirical antibiotic administration is initiated. The increasing rates of ESBL positivity and resistance to antibiotics such as ampicillin, cephalosporins, trimethoprim-sulfamethoxazole, and quinolones, especially in* E. coli *and *Klebsiella *strains, which are the most common pathological agents of UTI in our region, have limited the use of these treatments. However, high susceptibility of *E. Coli *growths to fosfomycin and nitrofurantoin and susceptibility of *Klebsiella *growths to gentamicin may make these antibiotics stand out as suitable options for the empirical treatment of UTI. Ensuring that hospitals apply the optimum empirical antibiotic treatment by identifying infectious agents and resistance patterns will provide the most effective treatment to patients and prevent the development of antibiotic resistance.

## References

[1] Dash M, Padhi S, Mohanty I, Panda P, Parida B (2013). Antimicrobial resistance in pathogens causing urinary tract infections in a rural community of Odisha, India. J Family Community Med.

[2] Tansarli GS, Athanasiou S, Falagas ME (2013). Evaluation of antimicrobial susceptibility of Enterobacteriaceae causing urinary tract infections in Africa. Antimicrob Agents Chemother.

[3] Foxman B (2010). The epidemiology of urinary tract infection. Nat Rev Urol.

[4] Zelikovic I, Adelman RD, Nancarrow PA (1992). Urinary tract infections in children. An update. West J Med.

[5] Sood S, Gupta R (2012). Antibiotic resistance pattern of community acquired uropathogens at a tertiary care hospital in Jaipur, Rajasthan. Indian J Community Med.

[6] Mazzulli T (2012). Diagnosis and management of simple and complicated urinary tract infections (UTIs). Can J Urol.

[7] Rizvi ZA, Jamal AM, Malik AH, Zaidi SM, Abdul Rahim NU, Arshad D (2020). Exploring antimicrobial resistance in agents causing urinary tract infections at a tertiary care hospital in a developing country. Cureus.

[8] Shaifali I, Gupta U, Mahmood SE, Ahmed J (2012). Antibiotic susceptibility patterns of urinary pathogens in female outpatients. N Am J Med Sci.

[9] Ağca H (2011). Bacteria isolated from urine samples and their antimicrobial susceptibilities (Article in Turkish). Kocatepe Medical Journal.

[10] Raka L, Mulliqi-Osmani G, Berisha L (2004). Etiology and susceptibility of urinary tract isolates in Kosova. Int J Antimicrob Agents.

[11] Uslu M, Bağcıoğlu M, Tekdoğan ÜY, Kocaaslan R, Çeçen K (2019). Epidemology and antibiotic resistance of urinary tract infection in the Kars region (Article in Turkish). Kafkas J Med Sci.

[12] Ünsal H, Kaman A, Tanır G (2019). Relationship between urinalysis findings and responsible pathogens in children with urinary tract infections. J Pediatr Urol.

[13] Kidwai SS, Nageen A, Ghaznavi S, Bashir F, Ara J (2017). Antibiotic susceptibility in commonly isolated pathogens from urinary tract infection in a cohort of subjects from low socioeconomic strata. Pak J Med Sci.

[14] Oladeinde BH, Omoregie R, Olley M, Anunibe JA (2011). Urinary tract infection in a rural community of Nigeria. N Am J Med Sci.

[15] Alzahrani MA, Ali MS, Anwar S (2020). Bacteria causing urinary tract infections and its antibiotic susceptibility pattern at tertiary hospital in Al-Baha region, Saudi Arabia: a retrospective study. J Pharm Bioallied Sci.

[16] Daoud N, Hamdoun M, Hannachi H, Gharsallah C, Mallekh W, Bahri O (2020). Antimicrobial susceptibility patterns of Escherichia coli among Tunisian outpatients with community-acquired urinary tract infection (2012-2018). Curr Urol.

[17] Akçam FZ, Gönen İ, Kaya O, Yaylı G (2004). The determination of susceptibility of beta-lactam antibiotics and extended spectrum beta-lactamase production in Enterobactericeae which responsible from nosocomial infections (Article in Turkish). Med J SDU.

[18] Yılmaz N, Ağuş N, Bayram A, Şamlıoğlu P, Şirin MC, Derici YK, Hancı SY (2016). Antimicrobial susceptibilities of Escherichia coli isolates as agents of community-acquired urinary tract infection (2008-2014). Turk J Urol.

[19] Topkaya AE, Kurç MA, Tombak Ö, Gülen D (2018). Investigation of extended spectrum beta-lactamase frequency and presence of carbapenemases in Enterobactericeae isolates growing in blood cultures. Namık Kemal Medical Journal.

[20] Gür D, Gülay Z, Akan OA (2008). Resistance to newer beta-lactams and related ESBL types in gram-negative nosocomial isolates in Turkish hospitals: results of the multicentre HITIT study (Article in Turkish). Mikrobiyol Bul.

